# COLOUR ATLAS OF LEPROSY

**Published:** 2008

**Authors:** P K Datta

**Affiliations:** *Department of Dermatology and STD, Professor and Head, Medical College and Hospital, Kolkata, India*

This colour atlas (meant for medical students, dermatologists, clinical leprologists and general practitioners) is a well written and comprehensive book containing a large number of clinical photographs that depict many colours of leprosy.

The author has included history, basic pathology, diagnostic modalities, clinical features, complications, differential diagnosis, and treatment of leprosy. Apart from these, two chapters on the unusual presentations and few unanswered questions have added flavour to this book.

The atlas would have been more appealing if it was devoid of a few unintentional typographical errors and unnecessary exhaustive inclusions of a few underexposed and ambiguous clinical photographs. There is a scope for improvement in the quality of the histopathological pictures.

In spite of this limited shortcomings, Dr. Ganguly must be congratulated for his sincere effort to compose this much needed exclusive atlas on leprosy, even in the post-leprosy elimination era.

I am sure that this will be useful for those for whom it is meant.

